# Development of Self-Healing Coatings Based on Linseed Oil as Autonomous Repairing Agent for Corrosion Resistance

**DOI:** 10.3390/ma7117324

**Published:** 2014-11-11

**Authors:** Karan Thanawala, Nisha Mutneja, Anand S. Khanna, R. K. Singh Raman

**Affiliations:** 1 IITB-Monash Research Academy, Powai, Mumbai 400076, India; E-Mails: khanna@iitb.ac.in (A.S.K.); raman.singh@monash.edu (R.K.S.R.); 2 Department of Metallurgical Engineering and Materials Science, Indian Institute of Technology Bombay, Powai, Mumbai 400076, India; 3 Amity Institute of Nano Technology, Amity University, Noida, Uttar Pradesh 201303, India; E-Mail: nishamutneja91@gmail.com; 4 Department of Chemical Engineering, Monash University, Victoria 3800, Australia; 5 Department of Mechanical & Aerospace Engineering, Monash University, Victoria 3800, Australia

**Keywords:** linseed oil, self-healing coatings, microcapsules, epoxy coating

## Abstract

In recent years corrosion-resistant self-healing coatings have witnessed strong growth and their successful laboratory design and synthesis categorises them in the family of smart/multi-functional materials. Among various approaches for achieving self-healing, microcapsule embedment through the material matrix is the main one for self-healing ability in coatings. The present work focuses on optimizing the process parameters for developing microcapsules by *in-situ* polymerization of linseed oil as core and urea-formaldehyde as shell material. Characteristics of these microcapsules with respect to change in processing parameters such as stirring rate and reaction time were studied by using optical microscopy (OM), scanning electron microscopy (SEM) and Fourier transform infrared spectroscopy (FT-IR). The effectiveness of these microcapsules in coatings was characterized by studying their adhesion, performance, and mechanical properties.

## 1. Introduction

The corrosion of metals is one of the most destructive processes that causes huge economic losses, in particular, to automotive, marine, oil and gas, and aerospace industries. Some of the efforts taken for its prevention are the use of alternative materials and design of components, and/or application of a suitable protective coating, depending on the type of environmental conditions, the metal is exposed to and the expected life. Among all of these, the most efficient and the most common approach to control corrosion is the application of organic polymer based coatings [1]. However, being the outermost layer on structures, these coatings are susceptible to damage and scratches originating at micro- and nano-levels during handling and service. Such damage is hard to detect, gradually propagating the corrosion process, and finally rendering the coating non-protective. The need is therefore to design and develop coatings which possess the ability to heal the damage thus maintaining the protective properties [2].

An active protection based on “self-healing” of defects in coatings is necessary for a durable effect. Active coatings with self-healing ability can be achieved from the intelligent release systems that are incorporated into the polymer matrix. Application of these coatings is a relatively recent concept in the corrosion protection technology. The active functionality is achieved via incorporation of “smart” release of micro-containers into the polymer. The micro-container can be tailored to trigger release through different mechanisms such as release under mechanical rupture, pH controlled release, ion-exchange controlled release or desorption controlled release [3]. Micro-containers loaded with a variety of functional materials have been used for different application fields such as biotechnology [4,5], preservation of flavors [6,7,8,9], sustainable drug release [9], electro-rheology fluids [10], dyes [11,12], and fire retardant powders [9,13,14].

There are different techniques available for encapsulation of reactive materials, which can be classified on the basis of a wall formation mechanism as reported by Pascault *et al.* [15]. Also, considerable literature is available on the synthesis methods and parameter optimization for encapsulation. The most important parameters that influence the size, shape, morphology, and thickness of shell of microcapsules are: stirring speed, type of emulsifier, concentration of emulsifier, pH, temperature and the duration of the encapsulation reaction [7,16,17,18,19,20].

In the present study, self-healing coatings, consisting of encapsulated drying oil for corrosion resistance have been investigated. Linseed oil was chosen as healing agent, the significance being its ability to form a film by oxidative drying [17,18,20,21,22,23,24,25,26,27]. However, the drying process can be accelerated by using driers, based on cobalt, calcium, lead, and zirconium. As a basis for further modifications and a useful model reference, the synthesis procedure, proposed by Wang *et al.* [19] was used. Critical process parameters such as stirring rate and reaction time were optimized for self-healing performance in corrosive environments. Also, adhesion and impact strength were characterized for examining the commercial feasibility of the coating.

## 2. Results and Discussion

For self-healing coating containing microcapsules, it is very important for the healing agent to remain intact within the shell, and get released easily when the microcapsules are ruptured. For both properties the shell formed should be thin and compact, which depends on the extent of the polymerization process. To investigate the influence of reaction time on shell thickness and extent of polymerization, the reaction time was varied between 1 and 4 h as shown in Table 1. In the synthesis of microcapsules, formation and stabilization of emulsion is very critical. This is greatly influenced by the concentration of emulsifier and the stirring speed. Thus the stirring speed was varied between 100 and 400 rpm as also shown in Table 1.

**Table 1:** Particle size and shell thickness data with change in reaction time and stirring speed.

Stirring Speed (rpm)	Reaction Time (h)			
	1	2	3	4
100	Particle size: 8 μm	Particle size: 10 μm	Particle size: 11 μm	Particle size: 13 μm
	Core size: -NA-	Core size: -NA-	Core size: -NA-	Core size: -NA-
	Shell thickness: -NA-	Shell thickness: -NA-	Shell thickness: -NA-	Shell thickness: -NA-
200	Particle size: 10 μm	Particle size: 13 μm	Particle size: 18 μm	Particle size: 17 μm
	Core size: -NA-	Core size: -NA-	Core size: -NA-	Core size: -NA-
	Shell thickness: -NA-	Shell thickness: -NA-	Shell thickness: -NA-	Shell thickness: -NA-
300	Particle size: 11 μm	Particle size: 14 μm	Particle size: 40 μm	Particle size: 45 μm
	Core size: 6 μm	Core size: 8 μm	Core size: 32 μm	Core size: 15 μm
	Shell thickness: 5 μm	Shell thickness: 6 μm	Shell thickness: 8 μm	Shell thickness: 30 μm
400	Particle size: 10 μm	Particle size: 12.5 μm	Particle size: 31 μm	Particle size: 35 μm
	Core size: 3 μm	Core size: 2.5 μm	Core size: 21 μm	Core size: 7 μm
	Shell thickness: 7 μm	Shell thickness: 10 μm	Shell thickness: 10 μm	Shell thickness: 28 μm

NA: Not applicable.

**Figure 1:**
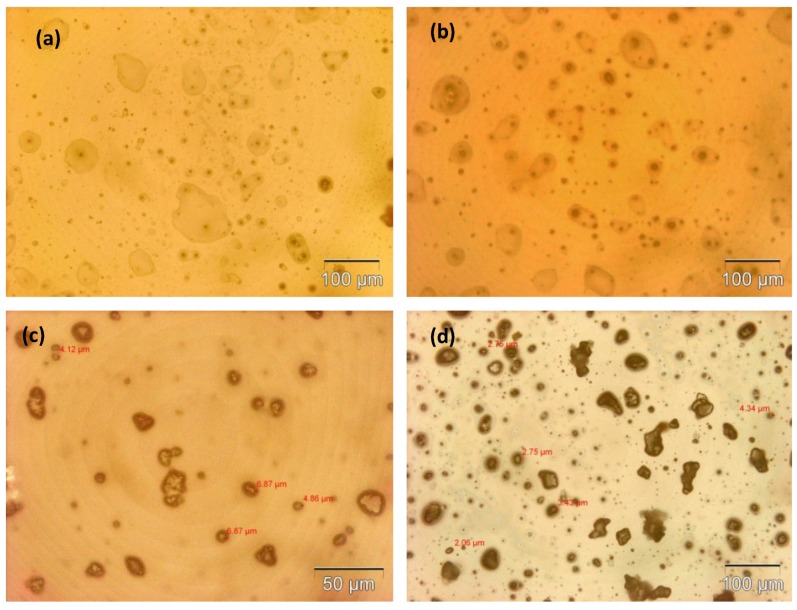
Optical microscopy images of microcapsules developed at a constant reaction time of 1 h but different stirring speeds: (**a**) 100 rpm; (**b**) 200 rpm; (**c**) 300 rpm; (**d**) 400 rpm.

**Figure 2:**
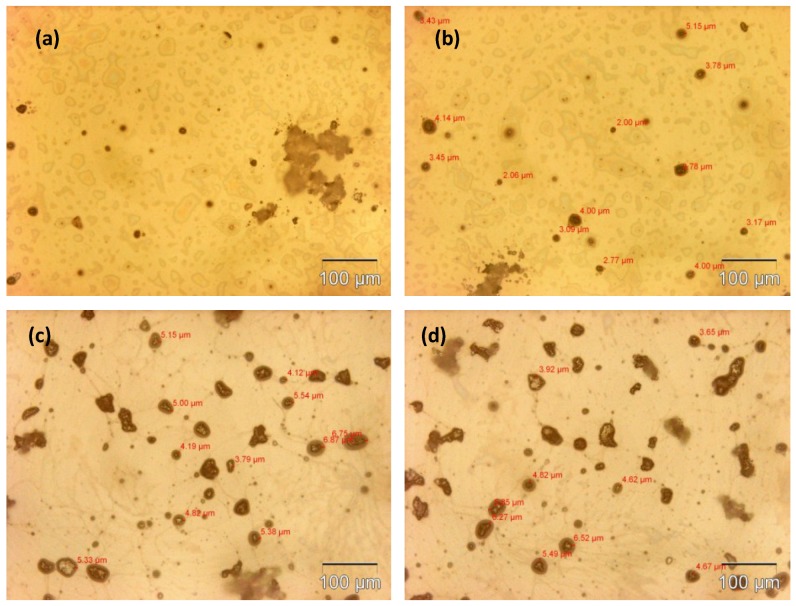
Optical microscopy images of microcapsules developed at a constant reaction time of 2 h but different stirring speeds: (**a**) 100 rpm; (**b**) 200 rpm; (**c**) 300 rpm; (**d**) 400 rpm.

**Figure 3:**
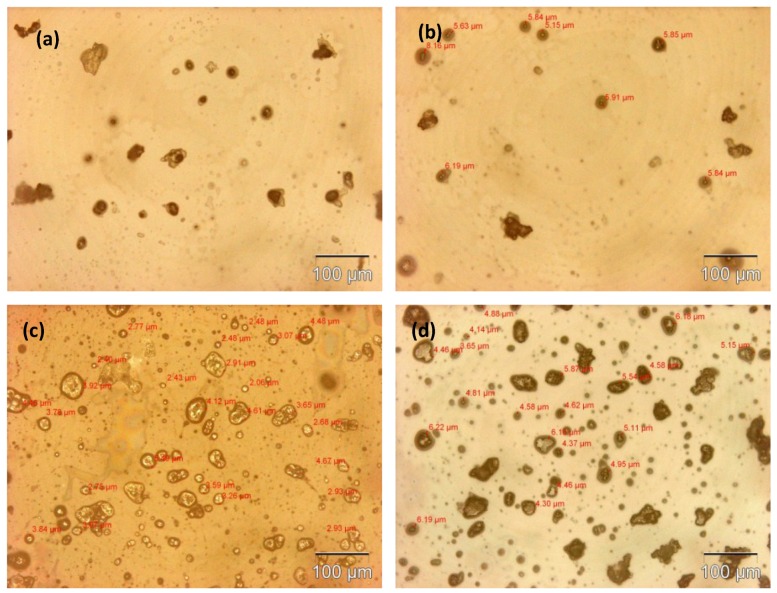
Optical microscopy images of microcapsules developed at a constant reaction time of 3 h but different stirring speeds: (**a**) 100 rpm; (**b**) 200 rpm; (**c**) 300 rpm; (**d**) 400 rpm.

**Figure 4:**
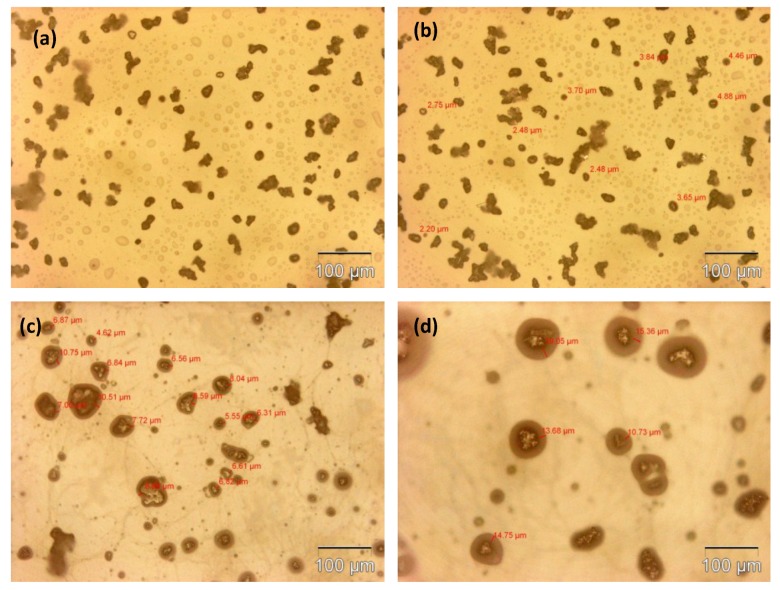
Optical microscopy images of microcapsules developed at a constant reaction time of 4 h but different stirring speeds: (**a**) 100 rpm; (**b**) 200 rpm; (**c**) 300 rpm; (**d**) 400 rpm.

The microcapsule mean diameter and shell wall thickness were determined using an optical microscope (Olympus GX51, Olympus Corporation, Shinjuku-ku, Tokyo, Japan) that was equipped with image analyzing software (Olysia m3, Olympus Corporation, Shinjuku-ku, Tokyo, Japan). Features in Figure 1a,b, Figure 2a,b, Figure 3a,b and Figure 4a,b show that the formation of urea-formaldehyde (UF) polymer particles predominated under the respective conditions, and microcapsule formation was negligible. The reason could be the stirring speeds of 100 and 200 rpm, which are insufficient for stabilization of the emulsion (which is also reflected in Table 1), leading to agglomeration of linseed oil forming a separate oil phase. The purpose of stirring is to form stable emulsion by breaking large oil droplets into smaller oil droplets. The agglomeration of oil droplets is favored at low stirring speeds, allowing formation of an unstable emulsion [23,28]. This affects the polymerization process of the urea formaldehyde shell around the oil droplets leading to formation of polymer particles. On the other hand, at stirring speeds of 300 and 400 rpm, the emulsion formed at the same dosage of surfactant was homogeneous and stable. Therefore, the formation of microcapsules was observed as shown in Figure 1c,d, Figure 2c,d, Figure 3c,d and Figure 4c,d.

As can be seen in the Figure 1c,d, Figure 2c,d, Figure 3c,d and Figure 4c,d and the summary in Table 1,the shell thickness increases with increasing reaction time, thereby leading to an increase in the permeability of the shell wall. One of the possible reasons which can be attributed to increased permeability is a facile polymerization reaction for shell formation at the interface in the direction normal to the surface of the core. This facilitated increase in the shell thickness [29]. However, with increase in thickness, the time required to complete cross-linking and polymerization of UF so as to form a compact-nonporous shell, also increases. From Figure 1, Figure 2, Figure 3 and Figure 4 and Table 1, it is evident that the combination of the reaction time of 3 h and the stirring speed of 300 rpm, allows formation of microcapsules with a maximum core having a thin and compact shell wall thickness. Further reaction leads to an increase in shell thickness and permeability.

**Figure 5:**
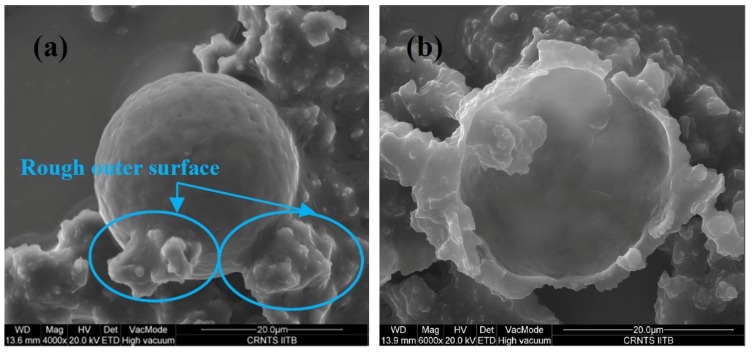
Scanning electron microscopy (SEM) images of microcapsules at 300 rpm and a reaction time of 3 h: (**a**) rough outer surface and (**b**) smooth inner surface.

The surface morphology of the microcapsules obtained at 300 rpm, and reaction time 3 h were investigated by scanning electron microscopy (SEM) (Hitachi S-3400N, High Tech Solutions Corporation, Tokyo, Japan). As can be seen, the microcapsule clearly had a rough outer surface (Figure 5a) and smooth inner surface morphology (Figure 5b). The rough outer surface morphology provides the additional interfacial area necessary for better adhesion with the film matrix. This probably eases the breakage of microcapsules due to stress generated in the scribed area [21].

**Figure 6:**
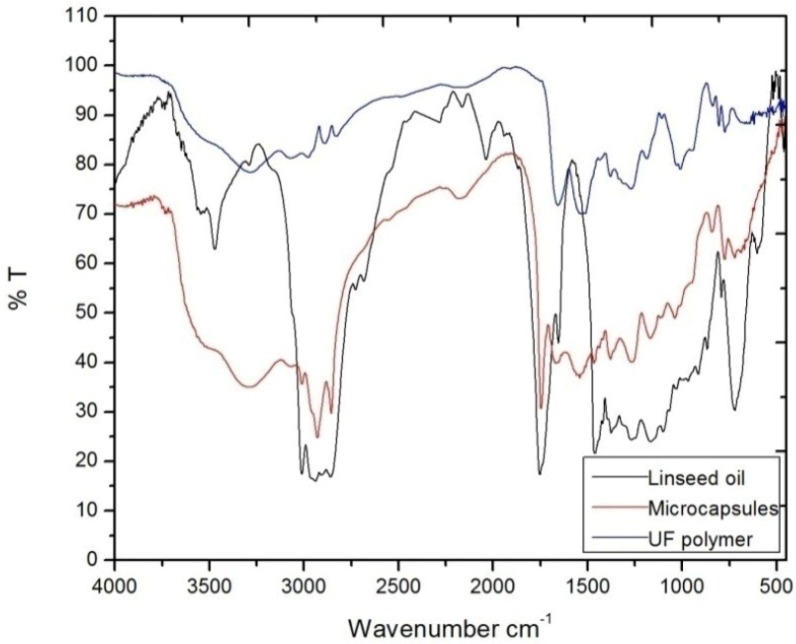
FT-IR spectra of microcapsules, linseed oil and urea-formaldehyde (UF) polymer.

Microcapsules prepared by an *in-situ* polymerization method under the optimized conditions were characterized using Fourier transform infrared spectroscopy (Jasco FT/IR 6100, Jasco, Easton, MD, USA) to confirm the presence of linseed oil and UF, the spectrum of UF having characteristic peaks of C–H stretching (2965 cm^−1^), N–H bending (1535 cm^−1^), C–H bending (1377 cm^−1^), C=O stretching (1656 cm^−1^), C–N stretching (1184 cm^−1^) and O–H stretching (3280 cm^−1^). The linseed oil’s spectrum having C–H stretching (2933 cm^−1^), C=C symmetric stretching (1654 cm^−1^), C=O stretching (1751 cm^−1^), C–H bending (1456 cm^−1^) and O–H (2924 cm^−1^) peaks. All the characteristic peaks of UF and linseed oil were found in the FT-IR spectrum of the microcapsule (Figure 6), which confirms the presence of UF and linseed oil in the microcapsule. Additionally, a shift of the C=C peak from 1654 to 1669 cm^−1^ of UF and the C=O peak of linseed oil from 1751 to 1741 cm^−1^ confirms the formation of cross linked polymer.

In view of the above, it is established that linseed oil has been successfully encapsulated in the UF shell. The oil content of the prepared microcapsules was determined for an extraction time of 5 h using the Soxhlet process as reported elsewhere [17,18,20,21,22,23,24,25]. The percentage linseed oil content in the microcapsules was determined to be 82% using the Soxhlet process.

### 2.1. Evaluation of Healing Performance

**Figure 7:**
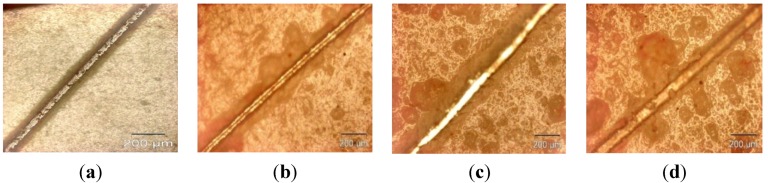
Self-healing ability of (**a**) blank epoxy coating and coating embedded with (**b**) 1 wt%; (**c**) 2 wt% and (**d**) 3 wt% of microcapsule (after 0 h).

**Figure 8:**
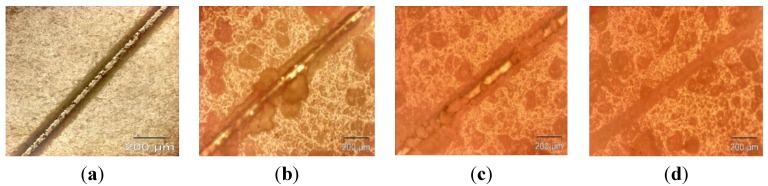
Self-healing ability of (**a**) blank epoxy coating and coating embedded with (**b**) 1 wt%; (**c**) 2 wt% and (**d**) 3 wt% of microcapsule (after 24 h).

**Figure 9:**
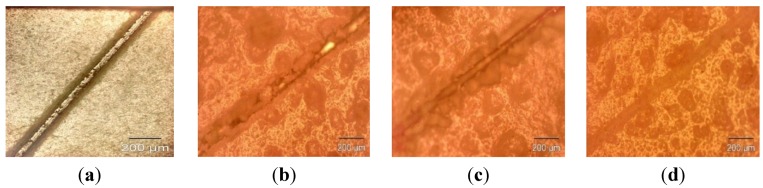
Self-healing ability of (**a**) blank epoxy coating and coating embedded with (**b**) 1 wt%; (**c**) 2 wt% and (**d**) 3 wt% of microcapsule (after 48 h).

**Figure 10:**
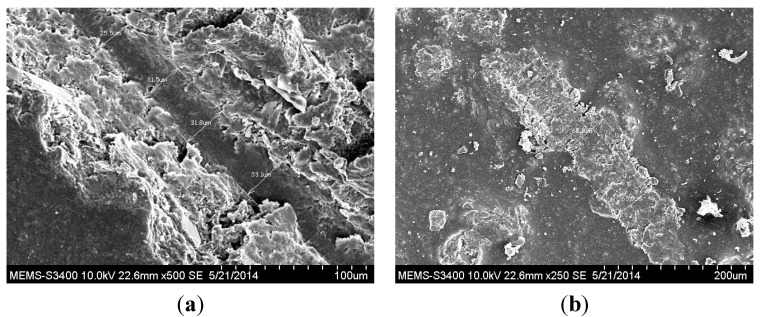
SEM images of the scribed region of the self-healing coating embedded with 3 wt% microcapsules (**a**) after 0 h of healing time and (**b**) after 48 h of healing time.

For preliminary evaluation of the mechanical integrity of the coating, the coating was scribed with the help of a sharp needle, after a curing period of 7 days. The self-healing ability in the coating is provided by linseed oil that is released from the rupturing of the microcapsules, which then fills in the cracks, further forming a film by oxidative polymerization of linseed oil in the presence of atmospheric oxygen and a combination of driers. The healing ability of the coating was examined for 48 h in a laboratory environment. The extent of healing for blank epoxy coating (without embedding microcapsules) and epoxy coating embedded with three different samples with varying microcapsule content (1%, 2%, and 3%) was observed using optical microscopy, as shown in Figure 7, Figure 8 and Figure 9. The coating loaded with 3% microcapsules healed seamlessly within 24 h, while the coating containing 2% microcapsules healed seamlessly in 48 h as shown in Figure 7c,d, Figure 8c,d and Figure 9c,d. In contrast, the coating with 1% microcapsules did not heal completely even after a period of 48 h, due presumably to the lack of healing agent available for seamless integration which is clearly visible from Figure 7b, Figure 8b and Figure 9b. However, it is evident from Figure 7a, Figure 8a and Figure 9a that no healing action can be seen in the blank epoxy coating. The reason can be attributed to the absence of healing agent (linseed oil) encapsulated in the synthesized microcapsules. The SEM Images of artificially scratched self-healing coating embedded with 3% microcapsules, at zero hours and after 48 h are shown in Figure 10. From the Figure 10a,b it can be confirmed that the scratch line had healed successfully. The coating was further characterized for its effectiveness of healing by means of immersion testing in a more corrosive environment.

### 2.2. Immersion Test

**Figure 11:**
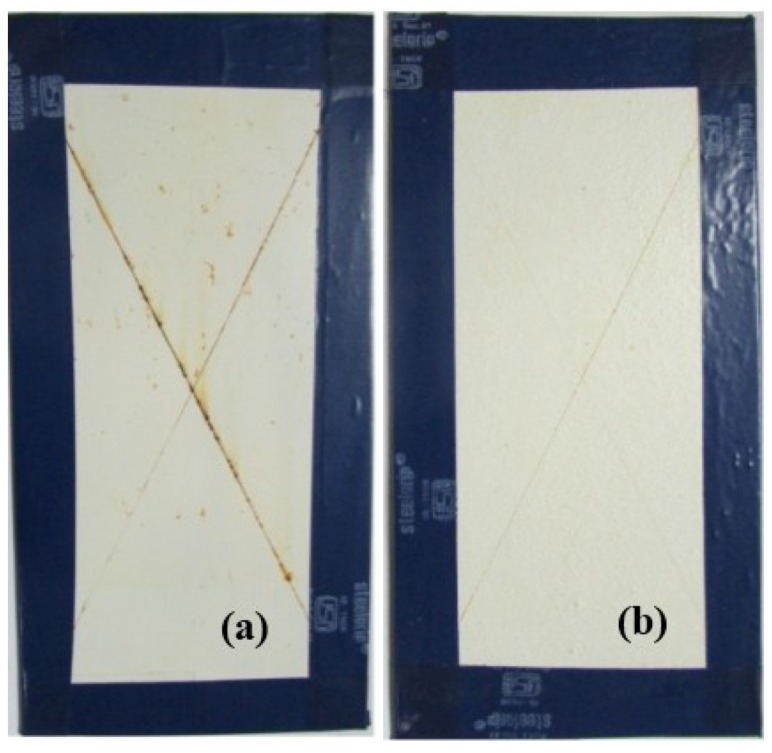
Coating surface after immersion in 3.5% NaCl solution after 200 h; (**a**) Control coating; (**b**) Self-healing coating embedded with 3% microcapsules.

**Figure 12:**
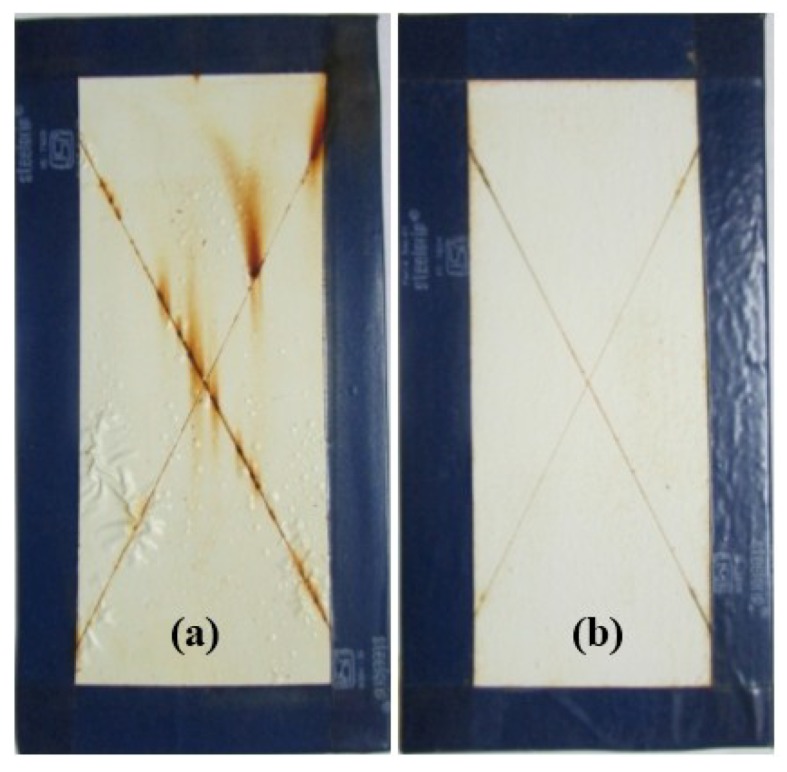
Coating surface after immersion in 3.5% NaCl solution after 500 h; (**a**) Control coating; (**b**) Self-healing coating embedded with 3% microcapsules.

To investigate the effectiveness of healing and the extent of corrosion protection offered by the self-healing coating, the control coating and the coating loaded with 3% microcapsules were immersed in 3.5% NaCl solution. The coatings were manually scribed with the help of a sharp needle to expose the underlying substrate to the corrosive environment. The control coating started forming blisters and rust within 24 h, and accelerated corrosion activity in the coming days. As shown in Figure 11a and Figure 12a, extensive rust formation was observed with increase in the duration of its exposure, particularly in the scribed region of the control coating. In contrast, the coating incorporated with 3% microcapsules showed no visible traces of blistering and rust formation over 200 h immersion (Figure 11b). Even over 500 h of immersion, only slight rust formation was observed in the scribed region (Figure 12b). This remarkable anticorrosive performance of the coating embedded with microcapsules, establishes the excellent ability of oxidative polymerization of linseed oil released from the ruptured microcapsules in healing the cracks, preventing further ingress of oxygen and moisture.

The adhesion test results (Table 2) show no difference in the mean adhesion values of the control and microcapsule embedded sample. Thus, it is evident that the incorporation of microcapsules did not result in any sacrifice to the adhesion.

The impact test results in Table 3 indicate no significant change in the resistance offered by the control sample and the coating embedded with microcapsules, and no significant loss of impact resistance.

**Table 2:** Adhesion strength of control and microcapsule embedded epoxy coated samples.

Sample	Adhesion strength of samples	Mean Adhesion strength
Control epoxy coating	5B, 5B	5B
Epoxy coating incorporated with microcapsules prepared at 300 rpm for 3 h	5B, 5B	5B

**Table 3:** Impact strength of control and microcapsule embedded epoxy coated samples.

Sample	Impact Strength of Samples (inch-lbs)	Mean Impact Strength (inch-lbs)
Control epoxy coating	233, 237, 243, 235, 240	237.6
Epoxy coating incorporated with microcapsules prepared at 300 rpm for 3 h	230, 238, 225, 233, 230	231.2

## 3. Experimental Section

**Figure 13:**
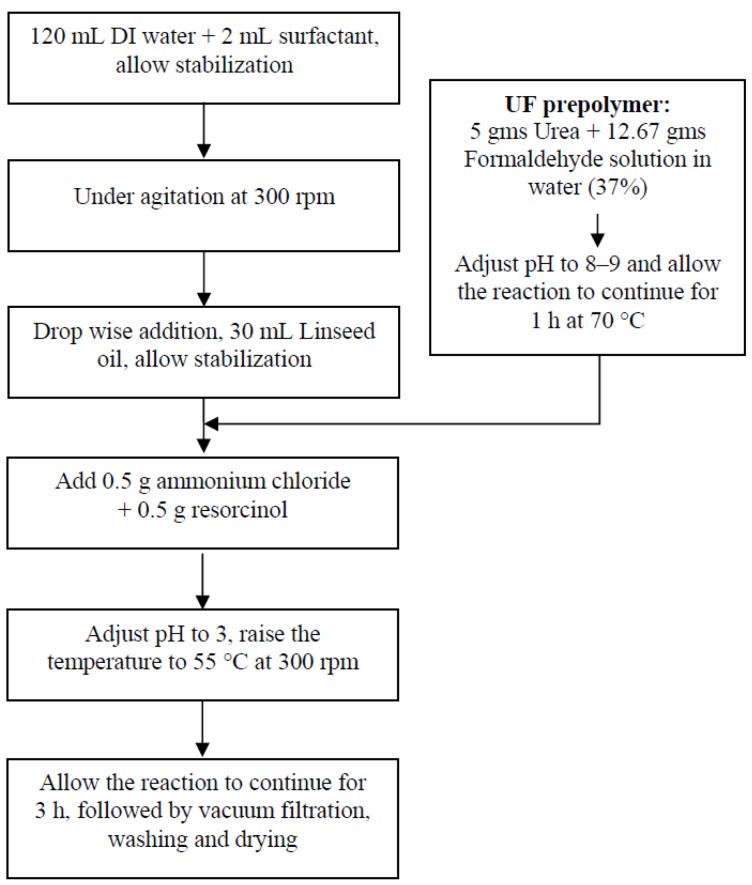
Schematic of the formation of microcapsules by two step *in-situ* polymerization.

The microcapsules were prepared by an *in situ* polymerization method using an oil-in-water emulsion technique [14,17,18,20,21,22,23,25,27]. The schematic of the process is shown in Figure 13.

### 3.1. Material

High purity materials required for forming microcapsule wall-forming materials, *viz.*, urea, formaldehyde (37 wt% formaldehyde in water) solution, ammonium chloride, resorcinol, triethanolamine (TEA), hydrochloric acid (HCl) were procured from Merck Co. (Mumbai, India), whereas linseed oil, surface active agent Fynol P, driers cobalt napthenate, zirconium octoate and the epoxy paint were procured from local sources.

### 3.2. Preparation of Pre-Polymer

Urea (5 g) and 12.67 g of formaldehyde were mixed in a 100 mL beaker, and stirred at 250 rpm until a clear solution was obtained. The pH of the solution was adjusted to 8–9, with TEA. The solution mixture was heated to 70 °C and held for 1 h, using a water bath. This procedure produced urea formaldehyde (UF) pre-polymer.

### 3.3. Synthesis of Microcapsules

An optimized mixture of non-ionic and anionic surfactant, Fynol P (2 mL) was added to 120 mL of deionized (DI) water in a 250 mL three necked flask. The mixture was stirred for 15–20 min, and then 25 mL of linseed oil was slowly added to form an emulsion. The emulsion was allowed to stabilize at 100–400 rpm for half an hour. After stabilization, UF pre-polymer was added to the emulsion at room temperature, followed by additions of 0.5 g resorcinol and 0.5 g ammonium chloride. After stirring for 20–30 min, the pH of the emulsion was adjusted to 2–3 by adding 5 wt% HCl. The solution was slowly heated to 50–55 °C, and the reaction was allowed to continue for 1–4 h, before allowing it to cool to ambient temperature. This procedure produced microcapsules that were recovered using vacuum filtration, and then washed thoroughly with DI water and xylene, to remove suspended oil particles and contamination. These microcapsules were vacuum dried at 50 °C. The microcapsules were in the form of a free flowing powder.

### 3.4. Encapsulation

**Figure 14:**
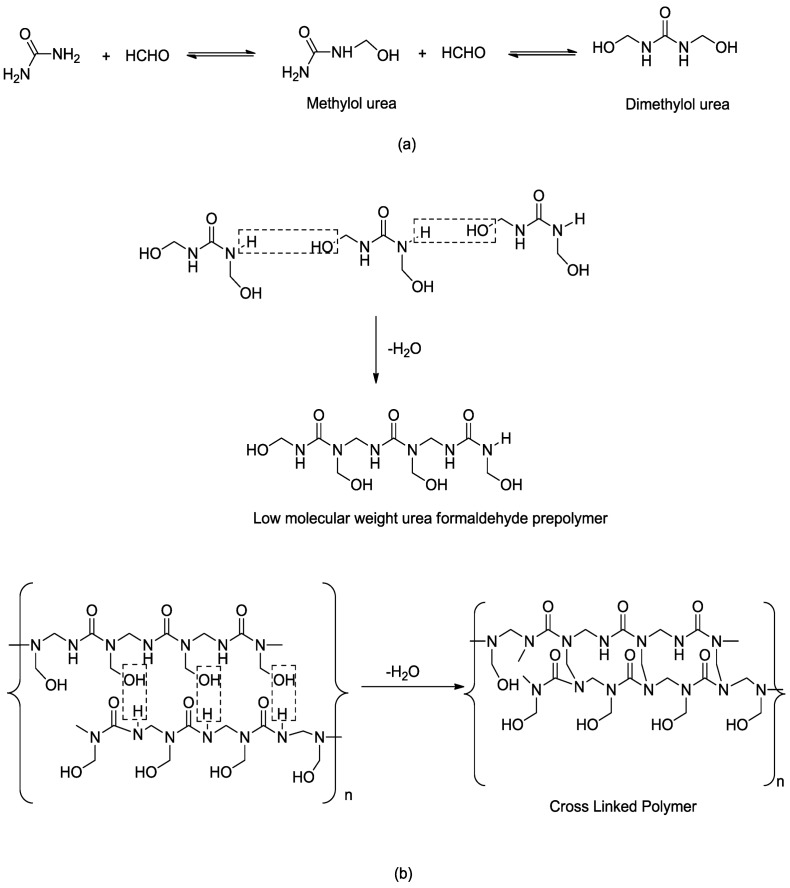
Chemical reaction of urea with formaldehyde form cross linked polymer.

The mechanism of *in-situ* polymerization reaction of urea formaldehyde (UF) microcapsules follows two steps. The first step, *i.e.*, addition reaction, involves the reaction between urea and formaldehyde, forming methylol urea as shown in Figure 14a. However, urea being tetra functional, the presence of excess of formaldehyde might lead to formation of tetra methylol urea. To restrict the formation of dimethylol urea, the pH of the mixture was adjusted to 8–9 and a temperature of 70 °C for 1 h. The reaction was carried out for 1 h. The second step involves condensation reaction between the molecules of dimethylol urea forming a low molecular weight pre-polymer. Further reaction leads to cross linked polymer as shown in Figure 14b. The reaction mixture was adjusted to pH 2–3 at 50–60 °C. The reaction speed can be controlled by varying temperature and pH, depending upon the extent of the desired cross-linking [30,31]. The addition and condensation polymerization reaction of urea formaldehyde is shown in Figure 13.

### 3.5. Determination of Linseed Oil Content in the Microcapsule

The linseed oil content of the microcapsule core was determined using the Soxhlet process [17,18,20,21,22,23,24,25]. The process follows a solvent extraction method, that is used to separate or isolate a species from a mixture of compounds or impurities, and that is based on solubility characteristics. Xylene was used as extracting solvent, owing to its good miscibility with linseed oil. A known weight of microcapsules (*W_i_*) was crushed using a pestle and mortar and transferred to a thimble. Pestle and mortar were rinsed with xylene and added to the thimble. After 5 h of extraction, at approximately 120 °C in an oil bath, the thimble was carefully taken out of the Soxhlet apparatus and after allowing the solvent to drain off completely, it was dried in an oven at 60 °C for 24 h. The final weight of the remained material (*W_f_*) was noted and the core content of synthesized capsules was determined using the following equation:

$$\text{Percentage linseed oil content of microcapsule}\;(\%)=\frac{W_{i}-W_{f}}{W_{i}}\times \;100$$

### 3.6. Preparation of Self-Healing Coating

To investigate the self-healing performance, epoxy based primer system with and without microcapsules was applied on commercially available CR1 grade mild steel substrate (3 inch × 6 inch × 0.0197 inch) as specified in IS 513 [32]. The substrates were first rinsed with acetone to remove contaminants such as oil and dirt, followed by cleaning with 200 mesh size grit paper. The microcapsules were incorporated into the epoxy based primer using two steps. In the first step, microcapsules were allowed to disperse homogeneously in 10 mL of solvent mixture comprising 30% xylene and 70% toluene by means of a water bath ultra sonicator for 30 min. After completion of the dispersion process, the microcapsules were mixed in the primer system using a three-blade mechanical stirrer for 20 min. The prepared self-healing coating was applied using a four-way applicator which gave a uniform coating thickness of around 50–60 microns (measured using Dry Film Thickness Gauge, Elcometer 456, Elcometer Instruments Ltd., Manchester, UK). For evaluation of the mechanical integrity and corrosion resistance of the coating, it was scribed with the help of a sharp needle, after curing for 7 days. The tip radius of the needle was 0.015 mm, made of stainless steel. The coating was scribed at a load of 500 g.

### 3.7. Adhesion Test

The probable negative effect of microcapsule embedment on the adhesion strength of epoxy coating was investigated by tape test according to ASTM D3359-09 [33]. The test was conducted using two control samples (without microcapsules) and microcapsule embedded coated samples respectively. The assessment involves applying and removing pressure sensitive tape over six cuts made in the film by means of a cross hatch gauge, spaced 2 mm apart.

### 3.8. Impact Test

Impact tests were conducted to determine the ability of a coating film to resist shattering, cracking, or chipping on rapid deformation, and thus to investigate the negative effect, if any, due to the microcapsule embedment on the mechanical property of the coating. The coated mild steel samples were tested according to ASTM D2794-93 [34], using 0.5 inch indenter diameter and 2 lbs indenter weight.

## 4. Conclusions

In the present study, various process parameters were successfully optimized to formulate a self-healing coating using linseed oil as healing material. The most important part of this work was the synthesis of microcapsules, which in turn depends upon various parameters such as stirring rate and reaction time. It was found that a stirring rate of 300 rpm and reaction time of 3 h resulted in microcapsules with optimum core and shell characteristics. The investigations confirmed that an epoxy coating incorporated with 3 wt% microcapsules, gave the best healing characteristics. No significant losses in adhesion and impact strength were identified as a result of the incorporation of microcapsules into the coating. Also, this coating showed remarkably superior resistance to corrosion-assisted blistering during 500 h immersion in 3.5% NaCl, as compared to a control sample having no self-healing constituent.
